# Genomic Sequence of the Strain Enterococcus faecium ICIS 18

**DOI:** 10.1128/MRA.01349-19

**Published:** 2020-01-09

**Authors:** M. V. Sycheva, L. P. Popova, T. M. Pashkova, Y. A. Khlopko, O. L. Kartashova, A. V. Andreeva, D. V. Poshvina

**Affiliations:** a Institute of Cellular and Intracellular Symbiosis of the Ural Branch of the Russian Academy of Sciences, Orenburg, Russian Federation; b Orenburg State Agrarian University, Orenburg, Russian Federation; c Bashkir State Agrarian University, Ufa, Republic of Bashkortostan; Indiana University, Bloomington

## Abstract

We report here the draft genome sequence of Enterococcus faecium strain ICIS 18, which was isolated from human feces. Analysis of the E. faecium ICIS 18 genome revealed genes encoding resistance to metals, fluoroquinolones, and beta-lactam antibiotics.

## ANNOUNCEMENT

Enterococci are widespread in the environment, and they are also representatives of the commensal microbiota of humans. Most of them synthesize various biologically active substances that have an inhibitory effect on pathogenic microorganisms (1, 2). Currently, it is of interest to search for promising probiotic strains of enterococci that do not have determinants of pathogenicity and have low sensitivity to antimicrobial agents (3).

The strain Enterococcus faecium ICIS 18 was isolated from the feces of a healthy person by using the classic bacteriological method. Feces were collected in sterile disposable sealed containers for further transportation to laboratory conditions. Next, their successive 10-fold dilutions in sterile isotonic NaCl solution were prepared. To dilutions 10-3 and 10-5, 0.1 ml of suspension was added to the surface of the following differential diagnostic media: Enterococcosel agar (Conda, Spain), bile-esculin agar with sodium azide (HiMedia, India), milk inhibitory medium (4), and a Drigalski spatula. Then, they were incubated in a thermostat at 37°C for 24 h.

Microorganisms were identified as species using multiplex PCR by the presence of species-specific genes encoding the synthesis of superoxide dismutase (5).

Total DNA was isolated from overnight broth culture using a combined method, including mechanical homogenization followed by enzymatic lysis. Briefly, 400 μl of tris-salt buffer (100 mmol/liter Tris-HCl, 20 mmol/liter EDTA, 750 mmol/liter NaCl [pH 8.0]) was added to 50 μl of the sediment of the test culture and homogenized using a TissueLyser LT instrument (Qiagen, Germany) with lyse matrix E (MP Biomedicals, USA) for 1 min at a frequency of 50 Hz. After that, 50 μl of tris-salt buffer with lysozyme (50 mg/ml) was added and incubated for 60 min at 37°C. Then, a 10% sodium dodecyl sulfate solution was added to the mixture to a final concentration of 1% with 2 μl of proteinase K solution (10 mg/ml), and it was incubated for 60 min at 60°С. After extraction with a mixture of phenol-chloroform-isoamyl alcohol (25:24:1) and subsequent extraction with chloroform-isoamyl alcohol (24:1), DNA was precipitated from the aqueous phase in 3 volumes of absolute ethanol with the addition of 10 M ammonium acetate (1:10) at −20°C overnight. After centrifugation and double washing with 80% ethanol, the DNA was dried and dissolved in 30 μl of deionized autoclaved water.

A DNA library was created using the Nextera DNA Flex library preparation kit in accordance with the manufacturer’s protocol. The size of the sequenced DNA fragment was 500 bp. Sequencing was carried out on the MiSeq sequencer (Illumina, USA) in the Center of Shared Scientific Equipment “Microorganisms Persistence” of the Institute of Cellular and Intracellular Symbiosis of the Ural Branch of the Russian Academy of Sciences using a MiSeq reagent kit v3 with 2 × 300-bp reads.

Sequencing data were analyzed using FastQC software v0.11.17. Based on this analysis and using Trimmomatic v0.36 (6) with the parameters LEADING: 30, TRAILING: 30, SLIDINGWINDOW: 30:30, CROP: 280, HEADCROP: 15, and MINLEN: 30, the original reads were cut off. The reads resulting from trimming were reanalyzed using FastQC v0.11.17.

The *de novo* genome was assembled using the genome assembler SPAdes v13.0 (7) with the –careful option.

The assembly yielded 279 contigs available in the NCBI GenBank with a total length of 2,733,006 bp, an *N*_50_ value of 60,802 bp, and an *L*_50_ of 12. It has a G+C content of 38.0% and an average coverage of 154.7×. The genome sequence was annotated using the Prokaryotic Genome Annotation Pipeline (PGAP) of the National Center for Biotechnological Information (NCBI) (https://www.ncbi.nlm.nih.gov/genome/annotation_prok), which identified 3,037 coding sequences, including 2,729 proteins, 216 pseudogenes, 5 rRNA genes (3 5S, 1 16S, and 1 23S), 69 tRNA genes, and 4 noncoding RNA genes (ncRNAs).

The strain is characterized by the presence of genes encoding resistance to copper, mercury, cadmium, cobalt, zinc, fluoroquinolones, and beta-lactam antibiotics, it has no genetic determinants of virulence (toxin formation, synthesis of superantigens), and it does not show pathogenic properties (gelatinase and hemolytic activity) on the phenotype, which makes the culture promising for use as the basis of a biological product of probiotic orientation.

### Data availability.

This whole-genome shotgun project has been deposited in GenBank under the accession number SWMT00000000. The version described in this paper is SWMT01000000. The raw sequence data are publicly available (SRA accession number SRR8983427).
